# Bromine and Its Preparatious in Disease

**Published:** 1868-02

**Authors:** J. J. Knott

**Affiliations:** Atlanta, Ga.


### ARTICLE II.

*Bromine and its Preparatious in Disease.* By J. J. Knott, M. D., of Atlanta, Ga.

There has perhaps no other class of remedies been so far overlooked by the medical profession as those at the head of this article. Having employed them extensively in my practice, with good results, I propose to give in a brief article a synopsis of my experience with them, more with a view of calling the attention of the medical fraternity to their employment, than any views that I am capable of giving, calculated to enlighten the profession as to their *modus operandi* upon the economy. I have found the preparations of Bromine as surely sedative in their effects upon the uterine system as ergot is a stimulant to the same, and have invariably employed them with marked success in the following affections: Dysmenorrhœa, hysteria, menorrhagœa, neuralgia of the uterus, and ulceration of the neck and mouth of the womb, all which we know to be attended with more or less excitement of the uterine system. An important advantage in the employment of these preparations, is that we can dispense with the employment of opiates, the pernicious effects of the continued use of which cannot be denied by any intelligent practitioner. Within the past month I have treated several cases of dysmenorrhœa with the bromide of potassium, all of which were relieved in from twelve to twenty-four hours. *I now have under treatment a case of ulceration of the mouth and neck of the uterus, of two years' standing. When I first saw the patient, some seven weeks ago, I found her suffering from the usual symptoms attending this affection, and, in addition, she experienced severe neuralgic pains, of such a severe nature as to deprive her of all rest, except that partially afforded by opiates. I placed her on 10 grain doses of the bromide of potassium, with a solution of muriate of ammonia, every four hours, 30 grains to the dose—cauterization every third or fourth day. She experienced immediate relief from the pain, and has continued to improve up to the present time, and I am confident that in two weeks time I can dismiss the case, entirely cured. Now without the controlling influence of the bromide of potassium over the irritation of the womb, I am satisfied that four months time at least would have been required to have effected a cure in this case, if it could have been effected at all under the ordinary treatment. I have not confined myself to females alone in the employment of these preparations, but have employed them successfully in epilepsy, chordee, spermattorrhœa, nocturnal emissions, and secondary syphilis. I usually commence with 6 grain doses, administered in the comp. S. of Sarsaparilla, repeated every three or four hours, increasing the dose gradually, if admissible. The following symptoms call for a diminution of the dose or its discontinuance: Giddiness, vomiting, severe pain along the spine, rigors, diarrhœa, etc. If by this article I can only induce some of the profession to give these pre-

* This case had been under the treatment of another physician of considerable reputation in the treatment of this affection, without any improvement following the ordinary reatment.

parations an impartial trial, I am satisfied that I will have accomplished some good for suffering humanity, as well as the medical profession.

